# Machine-Learning Studies on Spin Models

**DOI:** 10.1038/s41598-020-58263-5

**Published:** 2020-02-07

**Authors:** Kenta Shiina, Hiroyuki Mori, Yutaka Okabe, Hwee Kuan Lee

**Affiliations:** 1 Department of Physics, Tokyo Metropolitan University, Hachioji, Tokyo 192-0397 Japan; 2 Bioinformatics Institute, Agency for Science, Technology and Research (A*STAR), 30 Biopolis Street, #07-01 Matrix, 138671 Singapore, Singapore; 3 School of Computing, National University of Singapore, 13 Computing Drive, 117417 Singapore, Singapore; 4 Singapore Eye Research Institute (SERI), 11 Third Hospital Ave, 168751 Singapore, Singapore; 5 Image and Pervasive Access Laboratory (IPAL), 1 Fusionopolis Way, #21-01 Connexis (South Tower), 138632 Singapore, Singapore

**Keywords:** Information theory and computation, Phase transitions and critical phenomena

## Abstract

With the recent developments in machine learning, Carrasquilla and Melko have proposed a paradigm that is complementary to the conventional approach for the study of spin models. As an alternative to investigating the thermal average of macroscopic physical quantities, they have used the spin configurations for the classification of the disordered and ordered phases of a phase transition through machine learning. We extend and generalize this method. We focus on the configuration of the long-range correlation function instead of the spin configuration itself, which enables us to provide the same treatment to multi-component systems and the systems with a vector order parameter. We analyze the Berezinskii-Kosterlitz-Thouless (BKT) transition with the same technique to classify three phases: the disordered, the BKT, and the ordered phases. We also present the classification of a model using the training data of a different model.

## Introduction

Numerical simulations, such as Monte Carlo methods, have been successfully employed in the study of phase transitions and critical phenomena^1^. In spin systems, the spin configurations are sampled using a stochastic importance sampling technique, and the estimators for physical quantities, such as the order parameter and the specific heat, are evaluated for these samples.

Several spin models have recently been studied through machine learning^2–6^. Carrasquilla and Melko^2^ proposed a paradigm that is complementary to the above approach. By using large data sets of spin configurations, they classified and identified a high-temperature paramagnetic phase and a low-temperature ferromagnetic phase. It was similar to image classification using machine learning. They demonstrated the use of fully connected and convolutional neural networks for the study of the two-dimensional (2D) Ising model and an Ising lattice gauge theory.

In this study, we extend and generalize the method proposed by Carrasquilla and Melko^2^. First, instead of considering the spin configuration itself, we analyze the long-range correlation configuration, which will be explained later. From this analysis, we can evaluate the multi-component systems, such as the Potts model, and the systems with a vector order parameter, such as the XY model. We can identify identical configurations with the permutational symmetry or the rotational symmetry, which results in an efficient classification of phases. Moreover, the inclusion of long-range correlation is helpful in the study of phase transition. Second, we investigate the Berezinskii-Kosterlitz-Thouless (BKT) phase^7–10^, described by a fixed line instead of a fixed point from the perspective of the renormalization group, using the same treatment as the paramagnetic-ferromagnetic phase transition. By studying the 2D clock model, which is a discrete version of the XY model, we classify the paramagnetic-BKT-ferromagnetic transitions through machine learning.

## Model

We enlist the models we analyze below. We consider a 2D Ising model on the square lattice, whose Hamiltonian is given as

1 $$H=-\,J\sum _{\langle ij\rangle }\,{s}_{i}{s}_{j},\,{s}_{i}=\pm \,1.$$

The summation is realized over the nearest-neighbor pairs, and periodic boundary conditions are imposed in numerical simulations.

The Hamiltonian of the *q*-state Potts model^11,12^ is given by

2 $$H=-\,J\sum _{\langle ij\rangle }\,{\delta }_{{s}_{i}{s}_{j}},\,{s}_{i}=1,2,\cdots ,q,$$

where *δ*_*ab*_ is the Kronecker delta. The 2D ferromagnetic Potts model is known to exhibit a second-order phase transition for $q\le 4$ and a first-order phase transition for $q\ge 5$. The Potts model for $q=2$ is identical to the Ising model.

The 2D spin systems with a continuous XY symmetry exhibit a unique phase transition called the BKT transition^7–10^. A BKT phase of a quasi long-range order (QLRO) exists, wherein the correlation function decays as a power law. Here, we consider the *q*-state clock model, which is a discrete version of the classical XY model. Its Hamiltonian is given by

3 $$H=-\,J\sum _{\langle ij\rangle }\,\cos ({\theta }_{i}-{\theta }_{j}),\,{\theta }_{i}=2\pi i/q,\,i=1,2,\cdots ,q.$$

The 2D *q*-state clock model experiences a BKT transition for $q\ge 5$, whereas the clock model for $q=4$ comprises two sets of the Ising model and the 3-state clock model is equivalent to the 3-state Potts model. The clock model for $q=2$ is simply the Ising model.

We measure temperature in units of *J*.

## Correlation Configuration

The correlation function in the Ising model, with a distance *r*, is given by

4 $${g}_{i}(r)={s}_{i}{s}_{i+r}.$$

It clearly assumes a value of +1 or −1.

In the case of the *q*-state Potts model, the correlation function is defined by

5 $${g}_{i}(r)=\frac{q{\delta }_{{s}_{i}{s}_{i+r}}-1}{q-1}.$$

It assumes a value of +1 or $-1/(q-1)$.

The correlation function ${g}_{i}(r)$ of the *q*-state clock model is

6 $${g}_{i}(r)=\,\cos ({\theta }_{i}-{\theta }_{i+r}).$$

It assumes a value between +1 and −1.

There are several types of symmetries in spin systems. A few different spin configurations are essentially identical, whereas they have the same correlation configuration.

For phase transitions, it is preferable to include long-range correlations, which play an essential role in phase transitions. Because the longest distance in the finite-size systems of size *L* with periodic boundary conditions is *L*/2, we consider the average value of the *x*-direction and the *y*-direction, that is,

7 $${g}_{i}(L/2)=({s}_{x,y}{s}_{x+L/2,y}+{s}_{x,y}{s}_{x,y+L/2})/2,$$

for the Ising model. The same definitions are employed for other models.

We note that this type of correlation function was used along with the generalized scheme for the probability-changing cluster algorithm^13^.

Using the Swendsen-Wang multi-cluster flip algorithm^14^ for updating spins, we generated the spin configurations for a given temperature *T*.

The examples of the spin configurations {*s*_*i*_} and correlation configurations $\{{g}_{i}(L/2)\}$ for several models are shown in the Supplementary Information section. The plots of the 2D Ising model, the 2D 5-state Potts model, and the 2D 6-state clock model are shown in Fig. S1, Fig. S2, and Fig. S3, respectively.

## Machine-Learning Study

**Figure 1:**
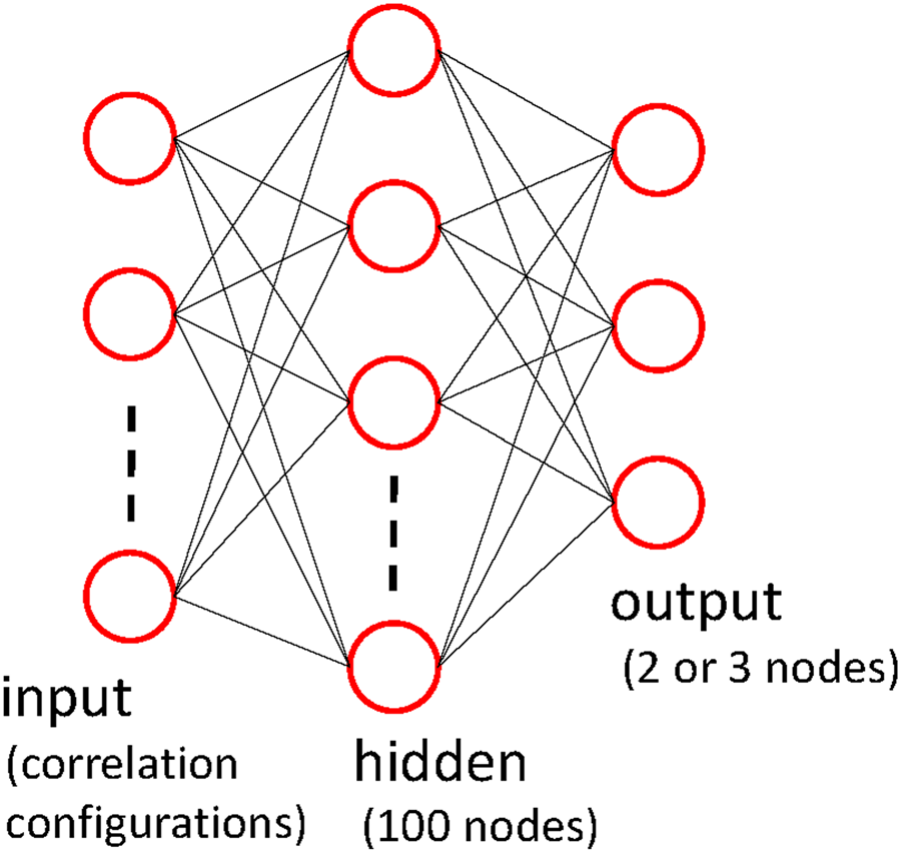
A schematic diagram of the fully connected neural network in the present simulation.

We have considered a fully connected neural network implemented with a standard TensorFlow library^15^ using the 100-hidden unit model to classify the ordered and the disordered phases. For the input layer, we use correlation configurations $\{{g}_{i}(L\mathrm{/2)\}}$. A schematic diagram of the fully connected neural network in the present simulation is shown in Fig. 1. We have used a cross-entropy cost function supplemented with an *L*2 regularization term. The neural networks were trained using the Adam method^16^.

Typically, around 40,000 training data sets are used, and 30,000 test data sets are used. Ten independent calculations were performed to provide error analysis. Although the exact transition temperatures *T*_*c*_ are known for most of the models in the present study, we have not used the samples close to *T*_*c*_ for the training data. We have assumed that the exact *T*_*c*_ is not known.

**Figure 2:**
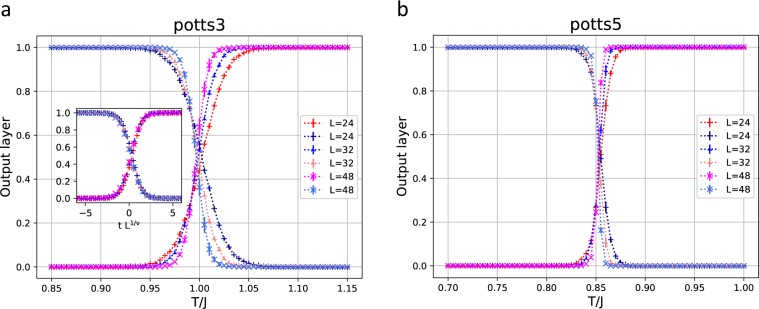
(**a**) The output layer averaged over a test set as a function of *T* for the 2D 3-state Potts model. The system sizes are *L* = 24, 32, and 48. The samples of *T* within the ranges $0.85\le T\le 0.94$ and $1.06\le T\le 1.15$ are used for the training data. In the inset, the finite-size scaling plot is given, where the horizontal axis is chosen as $t{L}^{\mathrm{1/}\nu }$ with $t=(T-{T}_{c})/J$. The values of *T*_*c*_ and *v* are ${T}_{c}=1/\,\mathrm{ln}(1+\sqrt{3})=0.995$ and *v* = 5/6, respectively. (**b**) The same plot for the 2D 5-state Potts model. The system sizes are the same. The samples of *T* within the ranges $0.7\le T\le 0.79$ and $0.91\le T\le 1.0$ are used for the training data.

We first analyzed the 2D 3-state Potts model. The output layer averaged over a test set as a function of *T* for the 2D 3-state Potts model is shown in Fig. 2a. The probabilities of predicting the phases, the disordered or the ordered, are plotted for each temperature. The system sizes are *L* = 24, 32, and 48. The samples of *T* within the ranges $0.85\le T\le 0.94$ and $1.06\le T\le 1.15$ were used for the training data. The exact second-order transition temperature *T*_*c*_ for this model is known as $1/\mathrm{ln}(1+\sqrt{3})=0.995$. We observed that the neural network could successfully classify the disordered and ordered phases. We give the finite-size scaling plot of the second-order transition^17^ in the inset, where the horizontal axis is chosen as $t{L}^{\mathrm{1/}\nu }$ with $t=(T-{T}_{c})/J$ and the correlation-length exponent *v*. For the values of *T*_*c*_ and *v*, we used the exact values, ${T}_{c}=1/\mathrm{ln}(1+\sqrt{3})=0.995$ and *v* = 5/6. We obtained very good finite-size scaling.

We have presented the output layer averaged over a test set as a function of *T* for the 2D 5-state Potts model in Fig. 2b. The system sizes are *L* = 24, 32, and 48. The samples of *T* within the ranges $0.7\le T\le 0.79$ and $0.91\le T\le 1.0$ were used for the training data. This model is known to exhibit the first-order transition at ${T}_{c}=1/\mathrm{ln}\,\mathrm{(1}+\sqrt{5})=0.852$. The transition is sharp compared with the Potts model for $q=3$ for the second-order transition.

**Figure 3:**
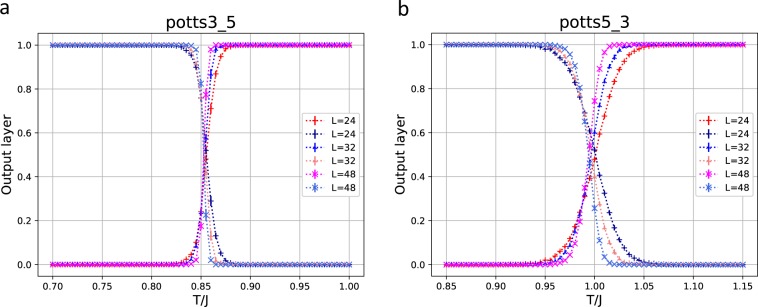
(**a**) The output layer for the 5-state Potts model using the training data of the 3-state Potts model. (**b**) The output layer for the 3-state Potts model using the training data of the 5-state Potts model.

It is instructive to use the training data obtained from the 3-state Potts model for the classification of the phases of the 5-state Potts model. The output layer for the 5-state Potts model using the training data of the 3-state Potts model is given in Fig. 3a. It successfully reproduces the sharp transition of the 5-state Potts model at ${T}_{c}=1/\mathrm{ln}(1+\sqrt{5})=0.852$. The plot of the opposite direction, that is, the output layer obtained for the 3-state Potts model using the training data of the 5-state Potts model is given in Fig. 3b. It reproduces the transition of the 3-state Potts model at ${T}_{c}=1/\mathrm{ln}(1+\sqrt{3})=0.995$. The order of the transition for the 3-state Potts model is second order, whereas that for the 5-state Potts model is first order. However, the training data of one model successfully reproduces the classification of the other model.

**Figure 4:**
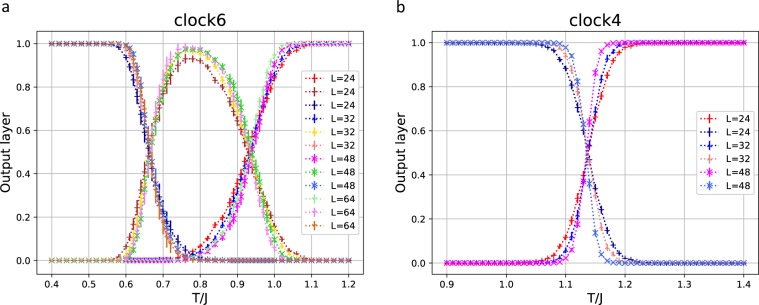
(**a**) The output layer averaged over a test set as a function of *T* for the 2D 6-state clock model. The system sizes are *L* = 24, 32, 48, and 64. The samples of *T* within the ranges $0.4\le T\le 0.64$, $0.77\le T\le 0.83$, and $0.96\le T\le 1.2$ are used for the training data. (**b**) The same plot for the 2D 4-state clock model. The samples of *T* within the ranges $0.9\le T\le 1.06$ and $1.2\le T\le 1.4$ are used for the training data.

We have considered the 2D *q*-state clock model next. Because of the discreteness, there are two transitions for $q\ge 5$. One is a higher BKT transition, *T*_2_, between the disordered phase and the BKT phase of QLRO, and the other is a lower transition, *T*_1_, between the BKT phase and the ordered phase. The recent numerical estimates of *T*_1_ and *T*_2_ for the 6-state clock model are 0.701(5) and 0.898(5), respectively^18^. The output layer averaged over a test set as a function of *T* for the 2D 6-state clock model is shown in Fig. 4a. The system sizes are *L* = 24, 32, 48, and 64. The samples of *T* within the ranges $0.4\le T\le 0.64$, $0.77\le T\le 0.83$, and $0.96\le T\le 1.2$ were used for the low-temperature, mid-range temperature, and high-temperature training data, respectively. Figure 4a shows the classification into the three phases. We estimate the size-dependent ${T}_{\mathrm{1,2}}(L)$ from the point that the probabilities of predicting two phases are 50%. The estimates of ${T}_{1}(L)$ and ${T}_{2}(L)$, in the range of $24\le L\le 64$, are around 0.66–0.67 and 0.93–0.94, respectively. The correlation length at the BKT transitions diverges rapidly, as given below,

8 $$\xi \propto \exp (c/\sqrt{|t|})$$

with $t=(T-{T}_{1,2})/{T}_{1,2}$, which is both below *T*_1_ and above *T*_2_. Finite-size effects result in a wider prediction of the BKT phase for smaller sizes. Size effects become smaller gradually with ln *L*. In the conventional Monte Carlo study of the BKT transition, the helicity modulus was calculated, and the size-dependent ${T}_{2}(L)$ can be estimated from the intersection with the straight line, $\mathrm{(2/}\pi )\,\ast \,T$, the universal jump^19,20^. The numerical estimates of ${T}_{2}(L)$ are 0.935 ($L=24$), 0.929 ($L=32$), 0.925 ($L=48$), and 0.921 ($L=64$), which slowly converge to 0.898 in the infinite *L* limit^18^. The present estimates of finite-size *T*_2_ are compatible with the universal jump analysis, although the systematic size dependence is hided because of statistical errors. The situation for *T*_1_ is the same. Thus, Fig. 4a clearly shows the behavior of the three phases.

It is interesting to investigate the relation between the BKT transition and the second-order transition. For this purpose, we have examined the 4-state clock model. This model is equivalent to two sets of the Ising model; it has a single second-order transition at ${T}_{c}=1/\mathrm{ln}(1+\sqrt{2})=1.135$. The output layer averaged over a test set as a function of *T* for the 2D 4-state clock model is given in Fig. 4b. The samples of *T* within the ranges $0.9\le T\le 1.06$ and $1.2\le T\le 1.4$ were used for the training data.

**Figure 5:**
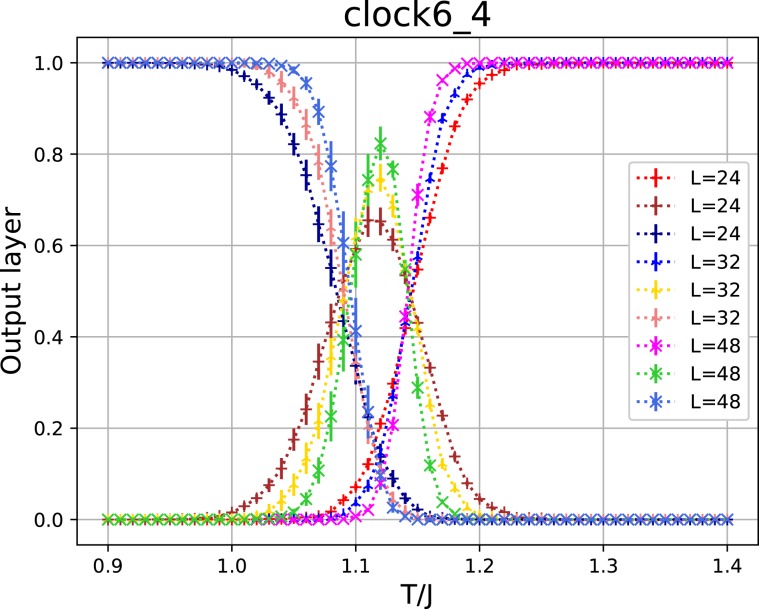
The output layer for the 4-state clock model using the training data of the 6-state clock model.

We investigate the result of using the training data of the 6-state clock model for the classification of the 4-state clock model. We present the output layer for the 4-state clock model as a function of *T* using the training data of the 6-state clock model in Fig. 5. The phases of the 4-state clock model are classified into the ordered and disordered phases with the expected *T*_*c*_ around 1.135. However, the narrow region near *T*_*c*_ is regarded as the BKT phase. It is an indication that the BKT phase with a fixed line is the same as the critical phase of the second-order transition with a fixed point. The figure indicates that the critical region becomes narrower as the system size increases.

## Summary and Discussion

We reported a machine-learning study on several spin models to study phase transitions. We considered the configuration of a long-range spatial correlation instead of the spin configuration itself. By doing so, we provided a similar treatment to various spin models including the multi-component systems and the systems with a vector order parameter. We successfully classified the disordered and the ordered phases, along with the BKT type topological phase. We showed a good finite-size scaling plot for the second-order transition.

Using the training data of the second-order transition system of the 3-state Potts model, we reproduced the phase classification of the first-order transition of the 5-state Potts model. The phase classification of the opposite direction was also successful. We achieved the phase classification of the second-order transition of the 3-state Potts model using the training data of the 5-state Potts model. Using the training data of the BKT transition system for the 6-state clock model, we elucidated the role of the critical phase of the second-order transition of the 4-state clock model. It is a direct demonstration that explains that the phase with a fixed line, whose spatial decay is an algebraic one, has the same structure as the critical phase of the second-order transition with a fixed point.

The present treatment of machine-learning study is generalized, and can be applied to various systems including quantum spin systems. It will be interesting to study the universal behavior of the topological phase of BKT type. There are sometimes implicit symmetries in the models of physics. Universality appears in totally different systems. The 3-state antiferromagnetic square lattice Potts model with a ferromagnetic next-nearest-neighbor interaction is an example. This model was studied by Otsuka *et al*.^21^ using the level-spectroscopy method, where they presented two BKT transitions and the universality of the 6-state ferromagnetic clock model. The machine-learning study on this model is in progress, and it is expected to be reported in the future.

## Supplementary information

Supplementary information.
