# Supplementary material for: Machine-Learning Studies on Spin Models
Source: Sci Rep. 2020 Feb 7;10:2177. doi: 10.1038/s41598-020-58263-5 (PMC7005704; doi:10.1038/s41598-020-58263-5)
Supplement: Supplementary file 1 — Supplementary information. [file 41598_2020_58263_MOESM1_ESM.pdf]

Machine-Learning Studies on Spin Models: Supplementary information

Kenta Shiina, Hiroyuki Mori, Yutaka Okabe, and Hwee Kuan Lee

Examples of the spin configurations and correlation configurations

As a supplementary information, we present examples of the spin configurations $\{s_i\}$ and correlation configurations $\{g_i(L/2)\}$, which is defined by Eq. (7) (main text). The 2D Ising model is displayed in Fig. S1. The spin configurations at the low temperature of $T = 2.0$, in units of J, are given in Figs. S1a and S1b. The ± 1 spins are displayed in red and blue. These two configurations are identical because of the inversion symmetry. The corresponding correlation configuration is shown in Fig. S1c. The correlations from $+1$ to -1 are mapped in gray scale from 255 (white) to 0 (black). The spin configurations at the high temperature of $T = 2.8$ are given in Figs. S1d-S1e, and the corresponding correlation configuration is given in Fig. S1f.

Examples of the spin and correlation configurations of the 2D 5-state Potts model are shown in Fig. S2. The five spin states are displayed in five colors, and there is a 120(=5!)-fold permutational symmetry in the 5-state Potts model. The spin configurations at the low temperature of $T = 0.8$ are shown in Figs. S2a-S2b, and those at the high temperature of $T = 1.2$ are shown in Figs. S2d-S2e. The spin configurations presented in Figs. S2a and S2b are identical, and the corresponding correlation configuration is given in Fig. S2c. The correlation configuration presented in Fig. S2f corresponds to the spin configurations presented in Figs. S2d-S2e. We note that the ordered-phase correlation configuration of the Ising model (Fig. S1c) and that of the Potts model (Fig. S2c) are similar. At the same time, the behavior of the disordered-phase correlation configuration of the Ising model (Fig. S1f) and that of the Potts model (Fig. S2f) are similar.

We have presented the spin and correlation configurations of the 2D 6-state clock model in Fig. S3. There is a 6-fold rotational symmetry. The spin configurations at the low temperature of $T = 0.5$, at the mid-range temperature of $T = 0.8$, and at the high temperature of $T = 1.2$ are given in Figs. S3a-S3b, S3d-S3e, and S3g-S3h, respectively. The corresponding correlation configurations are given in Figs. S3c, S3f, and S3i, respectively.

For all the models, the correlation configurations, shown in the third column of figures, exhibit similar behavior; they are almost white at low temperatures, whereas at high temperatures, they are mixtures of white, gray, and black.

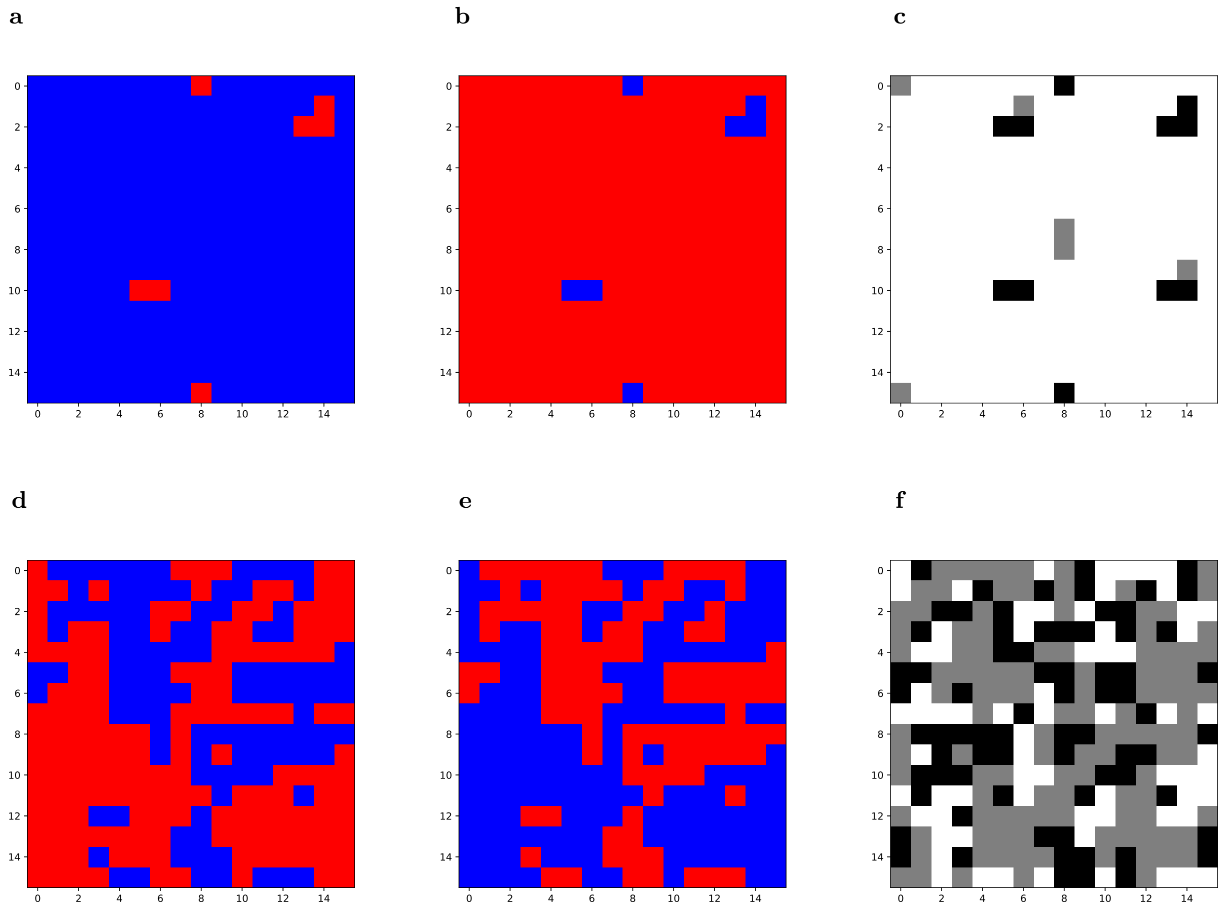

FIG. S 1: Examples of the spin configuration $\{s_i\}$ (a-b, d-e) and correlation configuration $\{g_i(L/2)\}$ (c, f) of the 2D Ising model. The upper figures (a-c) are snapshots at the low temperature of $T = 2.0$, and the lower figures (d-f) are those at the high temperature of $T = 2.8$.

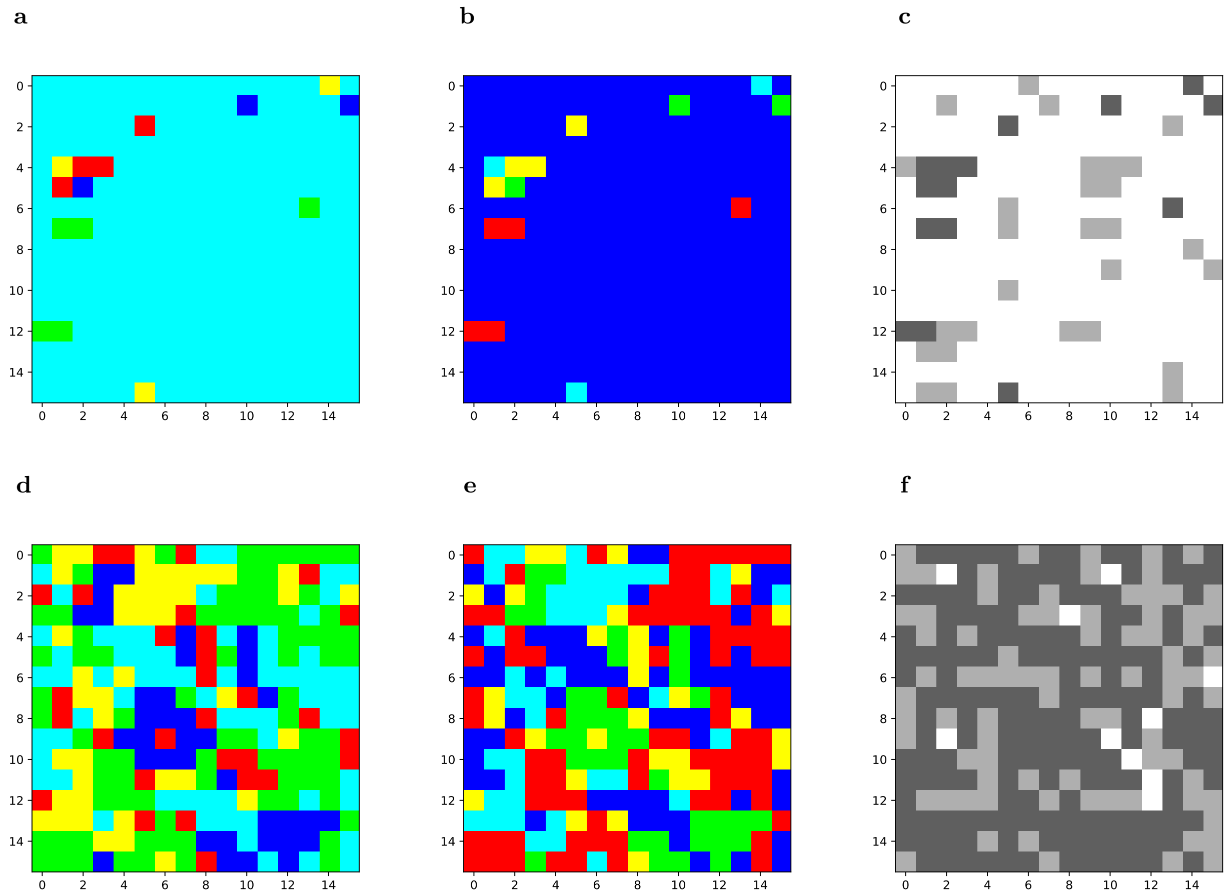

FIG. S 2: Examples of the spin configuration $\{s_i\}$ (a-b, d-e) and correlation configuration $\{g_i(L/2)\}$ (c, f) of the 2D 5-state Potts model. The upper figures (a-c) are snapshots at the low temperature of $T = 0.8$, and the lower figures (d-f) are those at the high temperature of $T = 1.2$.

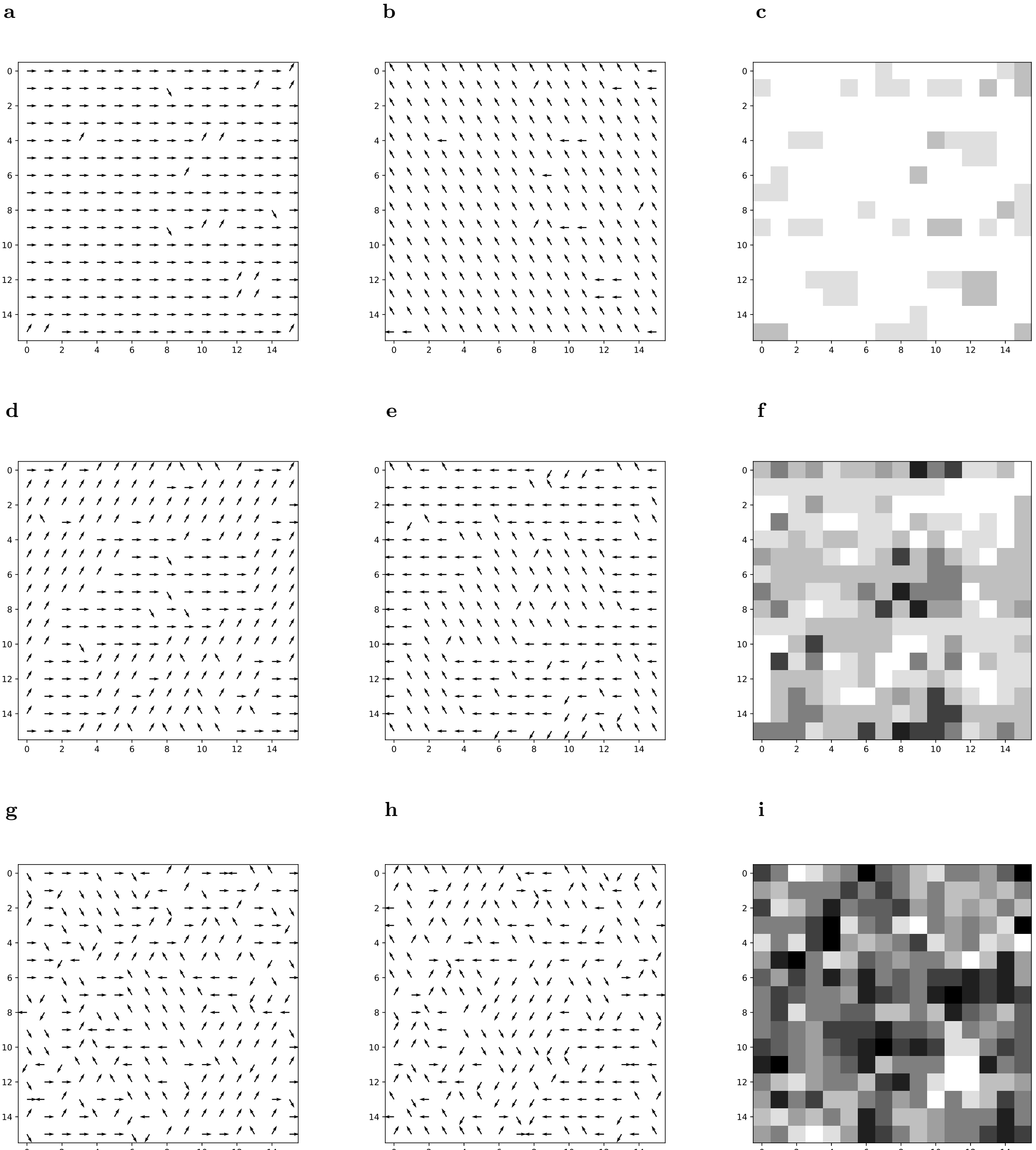

FIG. S 3: Examples of the spin configuration $\{\theta_i\}$ (a-b, d-e, g-h) and correlation configuration $\{g_i(L/2)\}$ (c, f, i) of the 2D 6-state clock model. The upper figures (a-c) are snapshots at the low temperature of $T = 0.5$, the middle figures (d-f) are those at the mid-range temperature of $T = 0.8$, and the lower figures (g-i) are those at the high temperature of $T = 1.2$.
